# Hyper-Spectral Imaging Technique in the Cultural Heritage Field: New Possible Scenarios

**DOI:** 10.3390/s20102843

**Published:** 2020-05-16

**Authors:** Marcello Picollo, Costanza Cucci, Andrea Casini, Lorenzo Stefani

**Affiliations:** Istituto di Fisica Applicata “Nello Carrara” del Consiglio Nazionale delle Ricerche (IFAC-CNR), Via Madonna del piano 10, 50019 Firenze, Italy; c.cucci@ifac.cnr.it (C.C.); a.casini@ifac.cnr.it (A.C.); l.stefani@ifac.cnr.it (L.S.)

**Keywords:** hyper-spectral imaging, Vis-NIR-SWIR imaging spectroscopy, non-invasive analytical technique, multivariate analysis, mapping materials

## Abstract

Imaging spectroscopy technique was introduced in the cultural heritage field in the 1990s, when a multi-spectral imaging system based on a Vidicon camera was used to identify and map pigments in paintings. Since then, with continuous improvements in imaging technology, the quality of spectroscopic information in the acquired imaging data has greatly increased. Moreover, with the progressive transition from multispectral to hyperspectral imaging techniques, numerous new applicative perspectives have become possible, ranging from non-invasive monitoring to high-quality documentation, such as mapping and characterization of polychrome and multi-material surfaces of cultural properties. This article provides a brief overview of recent developments in the rapidly evolving applications of hyperspectral imaging in this field. The fundamentals of the various strategies, that have been developed for applying this technique to different types of artworks are discussed, together with some examples of recent applications.

## 1. Introduction

In order to provide curators, scholars, conservators, archaeologists and conservation scientists with efficient tools for gaining knowledge of artifacts and archeological objects, it is important to study the materials and artists’ techniques used in creating the artworks and understand what restoration materials have subsequently been adopted in their preservation [1,2]. Presently, both invasive and non-invasive approaches can be used in the study of these materials; both have specific advantages and problems [2,3]. When dealing with cultural properties, the use of invasive techniques is discouraged since they require samples or micro-samples from investigated objects. However, these techniques offer more detailed information for identification purposes than non-invasive techniques. The latter, instead, are performed without sampling operations, and can be implemented as spot and as imaging techniques. Although they provide preliminary information on the materials and their distribution on the object’s surfaces, in most of cases, non-invasive techniques need a complimentary multimodal approach, in order to exhaustively characterize or identify all the materials. Usually, scientific investigation programs encompass a hierarchical use of analytical techniques, starting from non-invasive imaging techniques that are used for a preliminary screening and extensive evaluation of the surface, followed by non-invasive analytical spot techniques and, only when needed, as the last step, a complementary phase with micro-invasive techniques focused on investigating few suitable, selected points. Although not all these steps are always necessary, preliminary non-invasive analysis should always be recommended before starting any conservation or restoration procedure, in order to assist curators and conservators in their decision-making process. Non-invasive techniques, as such, do not alter the surface under examination, and are suitable for long-term or periodic monitoring of the same area. This approach is used to monitor the effectiveness of cleaning processes, restoration interventions, etc. In addition, monitoring over time is recommended in preventive conservation to safeguard artworks and prevent adverse actions of external agents (e.g., environmental agents, inappropriate microclimatic conditions, etc.).

When dealing with paintings, drawings, or other quasi-two dimensional (2D) art objects, imaging techniques are the preferred choice, not only for a preliminary evaluation of the conservation state of the artwork, but also for gaining knowledge on pigments and their distribution on the surface. Choosing the most suitable technique from the different available options depends on the size of the artwork, its value, the research interests and budget. The well-established traditional methodologies, such as ultraviolet-induced fluorescence (UVF), infrared reflectography (IRR), X-ray radiography (XRR) [4,5,6,7,8,9] are presently used together with more advanced techniques that enable the acquisition of multilevel information on pictorial materials, their distribution, and to some extent, also on their layering when suitable data-processing algorithms are used. These advanced imaging techniques include, multi-spectral imaging (MSI), hyper-spectral imaging (HSI), Macro X-Ray Fluorescence (Ma-XRF), and Time-Domain Terahertz imaging (THz-TDI), to name a few of the most significant [10,11,12,13,14,15,16,17,18,19,20,21].

This paper focuses on HSI, a cutting-edge technology that has nevertheless moved beyond the experimental phase, and has become quite accurate. It can be used in different applicative contexts, and can be implemented with several devices with different sensors and camera models. HSI devices for cultural heritage (CH) applications mainly operate in reflectance mode, and depending on the type of sensor, cover the Visible (Vis), Near Infrared (NIR), and Short-Wave Infrared (SWIR) regions. HSI enables the acquisition of a data-set that includes hundreds of spectral images acquired in very narrow spectral bands (bandwidth 2–10 nm). These data allow the reconstruction of a reflectance spectrum at each pixel in the scene, thereby, providing laboratory-like spectroscopic information useful for identification purposes. Algorithms that reduce the dimensionality of data and extract the required information are needed to process these data. Typically, image-processing is based on multivariate techniques, including statistical methods, such as Principal Component Analysis (PCA) and Maximum Noise Fraction (MNF), or automated classification methods, such as neural networks, t-Distributed Stochastic Neighbor Embedding (t-SNE) and Uniform Manifold Approximation and Projection for Dimension Reduction (UMAP) [22,23,24,25]. These methods allow the grouping and mapping of artists’ materials and alteration products according to their spectral similarities [12,26,27]. This provides elaborate images where the material’s distributions are mapped, thus, enhancing aspects that are not detectable by visual inspection. The capability of visualizing hidden details is further improved when the HSI data cover the SWIR region. In this case, investigations of the painted surface is extended to the inner layers of paintings, highlighting the underdrawings, *pentimenti*, etc. [15].

These various potentialities have encouraged further study of HSI usability as an advanced tool for responding at once to the needs of analysis, documentation and/or timely monitoring of different classes of polychrome surfaces. In particular, high-resolution reflectance spectra, obtainable at each pixel of the imaged surface, has rendered HSI data useful for colorimetric analysis and for a comparative evaluation of chromatic changes in time.

For over a decade, numerous studies and research projects have been dedicated to adapting image spectroscopy instrumentation to the specific needs of art preservation and conservation [12]. Specifically, HSI devices made by a few specialized companies or, in very rare cases, prototypes developed or readapted by research groups, have been applied on canvas, panel, paper, and parchment paintings in laboratory and museum contexts [15,28]. Typically, these devices have mainly been designed to work on panel paintings, at a macroscopic scale and at a short distance from the target, covering areas of limited sizes that range from tens of square centimeters to few square meters. However, recent publications have demonstrated that a remote sensing-like approach could successfully be used to extend HSI applications to larger surfaces, such as frescoes and mural paintings [29,30,31,32]. Such studies report on the use of readapted avionic HSI sensors, or in other cases compact HSI cameras featuring very flexible optical features that were used for the inspection of outdoors targets at distances of several meters.

Interestingly enough, very recently another research front has developed towards the opposite direction: To apply HSI for the study of small size and finely detailed art and historical objects, such as photographic materials, including contemporary color negative and positive films. One such occasion presented itself when a corpus of photographic materials, which was considerably damaged by a flood, was studied, restored, and digitalized within the Italian Tuscan Regional Project ‘*Memoria Fotografica*’ (Photographic Memory). The measurements acquired in this project were performed with a modified version of high-resolution spatial and spectral HSI scanner made by IFAC-CNR, which allows non-invasive operations on surfaces of variable dimensions. This innovative IFAC-CNR HSI prototype, used to study 35 mm photographic negatives and positives, was optimized for the inspection of details featuring millimetric (or sub-millimetric) sizes [33]. Due to the excellent spatial and spectral resolution of the developed system, this novel methodology is now suitable for being used in this specific sector to support the conservation and restoration of negative and positive films. To the best of the authors’ knowledge, it was the first time such a technique was applied to the analysis of negative films.

Another important application of HSI technique is related to archival high quality documentation of works of art. In this case, matching the requested requirements by curators and conservators involves dedicated HSI instrumentation and experimental protocols. These data have to be acquired with an illumination/observation geometry configuration that follows the *Commission Internationale de l’Eclairage* (CIE) recommendations, such as the 2 × 45°/0° or d/0° configurations, to provide calibrated RGB images and colorimetric values (i.e., CIEXYZ, CIEL*a*b*, sRGB, etc.) [34,35,36,37,38]. The application of HSI technique for the colorimetric analysis of paintings before and after the restoration is still rare in the field due to the difficulties in having accurate, reliable, and reproducible data suitable for matching the colorimetric calculations as required by CIE.

In addition to these two pioneering approaches, some significant examples of HSI applications in the CH field are presented in the following sections, with a focus on the most recent developments that point toward new applicative directions.

## 2. State-of-the-Art of HSI Systems

Imaging spectrometers collect data over three dimensions: Two spatial and one spectral. For this reason the acquired data-set is called file-cube (or data-cube, image-cube), as each item/datum is associated with two spatial coordinates (x, y) locating the pixel position, and with a spectral coordinate (λ), providing the reflectance intensity at each wavelength.

The most common method for categorizing the various types of imaging spectrometers is by the method of collecting the data-cube in a single detector readout; they are categorized as; (a) Push-broom spectrometers; (b) Staring; (c) Fourier Transform Imaging Spectrometry; (d) Whisk-broom Spectrometers; (e) Snapshot Imaging Spectrometers [39].

Currently, most of the HSI systems tailored for CH field are push-broom sensors. These systems generally use prism-grating (PG or PGP) spectrographs connected to Si CCD/CMOS (400–1000 nm), InGaAs (900–1700 nm), MCT, InSb sensors (1000–2500 nm).

To acquire the data set, the imaging system is pushed across the object under investigation (HSI scanner) or vice versa, and the image-cube is built up one spatial line at a time. Alternatively, the acquisition of the data can be done by using an internal device scan mirror and without moving the HSI device in front of the object (or vice versa) [12]. Push broom spectrometers use a focal plane array (FPA) detector, and thus collect a vertical slice of the data-cube at once so that only one spatial dimension needs to be scanned to fill out the cube.

In the staring systems, a filtered camera is constructed by placing a filter wheel or a tunable spectral filter in front of a camera. This device collects several images at different wavelengths and needs to take several images of the same detail to complete the data set [40,41,42].

Fourier Transform imaging spectrometry (FTIS) uses an internal interferometer, usually a Sagnac interferometer, which images the interference pattern onto the camera sensor [43,44]. This type of imaging systems are not common, particularly not in the Vis-NIR regions.

Whisk broom spectrometers, which use a linear array of detectors, collect a single column of the data-cube at a time and thus scan across the two spatial dimensions of the data-cube [12]. However, to the knowledge of the authors, this type of system is not as commonly used for CH applications.

Snapshot imaging spectrometers collect the entire three-dimensional (3D) data-cube in a single integration period without scanning. This imaging system is faster than the previous ones as it can take spectral images at real time (several frames per second), but to date, the spatial and spectral resolutions are poorer than, for instance, the push-broom systems [45,46].

## 3. HSI Application on Photographic Materials

Within the “Memoria Fotografica” (Photographic Memory, 2018–2019) project, recently funded by the Tuscan Region (Italy) and IFAC-CNR Florence (Italy), HSI technique was applied, for the first time, on photographic materials, namely on contemporary negative and positive films. This project had set as its primary objective the definition of a methodology for the recovery, stabilization and restoration of photographic collection of the “Dainelli archive”, which was damaged by the flood that hit the territory of Leghorn, Italy, in September 2017. This archive included different classes of photographic material (color negatives, color slides, prints with chromogen and inkjet development) that were severely compromised by this extreme event. HSI technique was selected as an innovative scientific approach that could simultaneously face multiple needs, including: (a) analysis of the photographic materials (dyes and emulsions); (b) digitization; (c) tentative digital restoration and/or recovery of lost parts. Since the use of HSI for investigation on photographic negatives was unexplored until this pilot project, the instrumentation had to be re-designed for this specific purpose. At IFAC-CNR laboratories, a readapted version of the HSI scanner [47,48], operating both in transmission- and reflectance-mode, was devised to inspect negative and positive photographic films.

The hyper-spectral scanner for measurements in transmission on photographic material consists of a linear spectrograph connected to a camera, a mechanical part that allows the movement of photographic film pieces, a projector that uniformly illuminates a diffusing screen positioned almost in contact with the sample to be measured. The composed instrument is connected to a computer that allows its management and provides the acquisition of data and their display.

With this instrumental configuration, the scanner operates in the 400–900 nm range. The measuring head is equipped with an Opto-Engineering TC23024 telecentric lens, with a Prism-Grating type transmission spectrograph (Specim ImSpector™ -Oulu, Finland- mod. V10E) and with a Hamamatsu digital camera CCA ORCA–ERG model.

Scanning is obtained by moving the target (negative*/*positive film) with horizontal streaks up to about 25 cm. Prior to each horizontal scan, a calibration is performed on the illuminant itself. Lighting is provided by a 150 Watt tungsten-quartz halogen lamp (QTH) with a color temperature of approximately 3200 K. The instrument operates with a spatial sampling step of about 37 microns, resulting in the acquisition of approximately 27 points per millimeter, equivalent to almost 700 ppi. Thus, the obtained spectral images have, at half of the maximum contrast, a spatial resolution of 5 lp/mm. The spectral sampling is performed in steps of about 1.2 nm, which produces 400 bands and results in a spectral resolution of approximately 2.8 nm in the operating range.

Presently, scientific analysis applied to photographic films and their constituent materials for conservative purposes are quite fragmentary, and there are hardly any publications available dealing with this matter [49,50,51,52].

**Figure 1:**
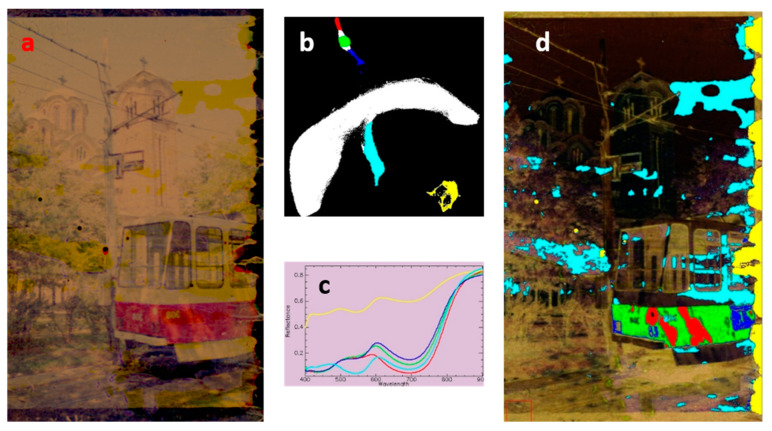
(**a**) sRGB image of the investigated frame; (**b**) UMAP image with the selected five sub-sets of fairly distinct pixels (highlighted in red, green, blue, cyan, and yellow); (**c**) spectra extracted from the data-cube representative of the UMAP five classes reported in (**b**); (**d**) displacement of the UMAP five classes on the HSI reconstructed visible image.

From a material point of view, the film system, as far as the color is concerned, can be considered relatively simple, with a three-layer structure. Each layer contains a specific dye, namely yellow, magenta and cyan dyes, to which other two correcting dyes have to be added. However, due to the complex and extensive gamut of color offered by the negative and positive color films, in the 400–900 nm range the identification of these colorants is far from easy. In fact, working in transmittance the three layers contribute to the final spectra producing complex spectral features (absorption bands) that are quite similar on all the pixels [52]. This makes it difficult attaining an analytical information and identification of dyes formulation (e.g., based on their commercial brand). However, it is possible to process the HSI data in order to infer some knowledge on the state of conservation of the frames, and thus to support with accurate spectroscopic information the digitalization phase and the subsequent digital restoration of the photographic material. In some cases, even partially lost parts of a scene can be reconstructed by recovering the information on colors from preserved details. As an example of the potentialities of this type of analysis, the results obtained on a color negative photogram, showing dented and un-homogeneous areas, are reported in Figure 1.

Figure 1a shows the entire sRGB image reconstructed from the HSI data of a negative color that had remained attached to the glassine envelope following the flood (after being in the water and mud for nearly two weeks) and detached during the restoration intervention.

To optimize the spectroscopic study of these frames, automated classification methods were used on these image-data in order to obtain a separation in homogeneous areas, or rather, to group pixels in classes that shared similar spectral trends. This phase of the research focused on testing a series of algorithms and procedures to define these areas. Among the various algorithms tested, a new statistical approach was proposed based on a mathematical model called “UMAP” (Uniform Manifold Approximation and Projection for Dimension Reduction) [25]. UMAP is a scalable algorithm for data dimension reduction that favors the preservation of local distances over global distance. It is built upon mathematical foundations related to the work of Belkin and Niyogi on Laplacian eigenmaps [53,54]. In the UMAP procedure, the elements (pixels) that make up the region of interest (ROI) are grouped based on the similarity of their spectral trend (Figure 1b). In this map, lightest areas correspond to the most represented areas. In the distribution map there is a rather compact central body (reported in white), while a peninsula and two detached areas are distinguishable in its periphery. To recognize the pixels on the sRGB image grouped by spectral similarity in these areas, an arbitrary color has been assigned to these zones (or classes). In Figure 1b five classes of pixels are highlighted in red, green, blue, cyan, and yellow. The corresponding average transmittance spectra, representative of these classes, are shown in Figure 1c. It can be observed that the classes represented with red, green, and blue colors have similar spectral trends, which are mainly dominated by spectral features of the magenta color present in part of the tram body (Figure 1d). The spectral differences in these three classes are instead related to the darker tone of the portion of the tram (blue curve), to the areas that are partially overshadowed by the trees (green curve) and to the remaining part under the sunrays (red curve). The red area in Figure 1d could also be related to the portion of the frame that was damaged by water. The other two classes, cyan and yellow, map the distribution in the frame sections that are lightly and severely damaged, respectively. The yellow class, particularly, groups pixels with spectra that show weak spectral features originating from the chromatic emulsions of the film during the harsh washing process during and after the flood.

Strengths and weaknesses UMAP algorithm are summarized in [25]. In particular, in Section 6 it is remarked that whenever the interpretability of the reduced dimension results is of critical importance, algorithms like PCA must be preferred with respect to t-SNE and UMAP. Nevertheless, methods like t-SNE and UMAP tend to keep the local structure with respect to the global structure of data, which in HSI application means that pixels that contribute to different clusters not necessarily have so distant spectra as the distance among the clusters may suggest. Here, to the authors’ knowledge, UMAP resulted to be better scalable than t-SNE, which means that its results depend less on data dimensions. On IFAC-CNR data, UMAP has worked well up to about 7,000,000 spectral pixels. To give an idea of the speeds, UMAP processes 429,165 pixels with 399 bands in 857.47 s, while t-SNE version optimized for Multicore CPUs by Dmitry Ulyanov (https://github.com/DmitryUlyanov/Multicore-TSNE) employs 2905.28 s.

As regards to the digitalization and digital restoration, HSI can help detect whether the dyes of the emulsions have maintained their chromatic characteristics or have undergone alterations based on the transmittance spectra extracted from the cube-file. These data need to be complemented with the technical data reported by the producers of photographic film. To this aim, HSI data-analysis based on PCA (Principal Component Analysis) demonstrated to be effective. Specifically, PCA was successfully applied to reconstruct the absorbance curves of each individual dye from their mixtures [52]. Using this procedure, the absorption curves of the three basic layers could be obtained for each frame, or groups of frames, to be compared with the data provided by the manufacturer when the films were produced/purchased. After an iterative process, the reported procedure converges toward an output data set that can be used to guide the digitalization of the degraded altered frames and their subsequent digital restoration. These preliminary results indicate that HSI can play a prominent role in the long-term preservation and development of photo inventory in archives and image collections [55].

## 4. HSI Application for Colorimetric Analysis of Paintings

Non-invasive diagnostics as well as accurate color acquisitions on paintings are possible with the HSI scanner developed at IFAC-CNR. Its orthogonal pair of linear motion actuators move a compact line-spectrograph in a vertical-plane. The adjacent vertical strips, which slightly overlap, perform the actual scan movement. The Specim Ltd. spectrographs ensure geometrical deformations that can be considered negligible, while the additional filters compensate any internal stray-light. This system’s spectral range is in the 400–1650 nm, with two separate spectrographic heads covering the VNIR (400–900 nm), which is also used for colorimetric applications, and SWIR (950–1650 nm) ranges with a spectral resolution of 2.5 nm and 8 nm, respectively. The devices are pushbroom HSI models based on PGP spectrographic heads. The technical details have been previously described in [47,48].

A fiber-optic illuminator (Schott-Fostec) which directs the radiation of a 3300 K QTH lamp onto a fiber-optic bundle forms the basis of the illumination module that terminates with a pair of light-lines with cylindrical lenses. The geometry of the illumination system is conformed to CIE standards for colorimetric measurements, projecting their beams around the viewed line-segment, symmetrically at 45° with respect to the surface [47]. The internal stray-light is one of the main cause of colorimetric errors that can be compensated only by a flat subtraction from all the wavelengths of the signal measured on the spectral channels below 400 nm.

The magnification of details without compromising quality is possible due to the combination of high color accuracy and high spatial sampling (0.1 mm) obtained from the image-cubes. Thus, it is possible to visualize, for instance, the pattern of paint cracks (*craquelure*), which is a detail of great interest in conservation documentation [38].

As a case study, the analysis of the “*Polittico dell’Intercessione*” (ca. 1425), a panel with five partitions (97 cm × 222 cm), painted by Gentile da Fabriano (c. 1370–1420), in the Church of San Niccolò Oltrarno in Florence, is reported. The HSI measurement was mainly focused on a color evaluation of the paint surface before and after the challenging restoration operations. These five partitions depict Saint Louis of Toulouse, the Resurrection of Lazarus, the scene of the intercession of Christ and the Holy Virgin with God, Saints Cosma and Damian, and also Saints Julian and Bernard [56]. It was considered a cryptic, unreadable work until an important restoration project made it comprehensible. The first documentation on this work, dated 1862, reported that the painting belonged to the Florentine church of San Niccolò Oltrarno. In 1897, the panel was badly damaged by fire, which magnified the alterations induced by previous restoration. Therefore, the polyptych was considered to be irrevocably damaged and, consequently, was stored for decades, until 1995, when it was selected for a new restoration project carried out by the *Opificio delle Pietre Dure* (OPD) in Florence, Italy [56].

**Figure 2:**
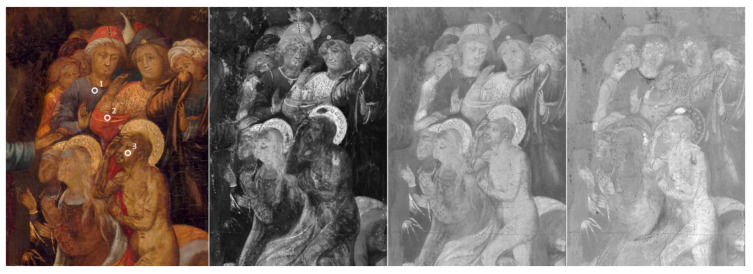
From left to right: sRGB of the Lazarus detail reconstructed from the HSI data at the end of the restoration procedure with the spots whose colorimetric values are reported in Table 1; images in grey scale of the b* colorimetric values before restoration; after being restored; image difference: **∆**b* colorimetric values (after–before).

Due to a standardized experimental acquisition protocol, that includes reproducibility of illumination, data-acquisition, repositioning, etc., IFAC-CNR HSI device measurements guarantee comparability between different measurements sessions. In this specific case, it was possible to colorimetrically compare the images collected before and after the restoration procedures on the painting. These data were stored as CIEL*a*b*76 TIF images files; it was possible, therefore, to visualize the three-color parameters (L*, a*, b*) as three separated grey level maps, in which high values for the three parameters corresponded to brighter pixels in the resulting image file. In Figure 2, the reconstruction of a detail of the scene “the Resurrection of Lazarus” is reported. Here, the sRGB image reconstructed from the HSI data at the end of the restoration procedure is reported together with the images of the b* colorimetric values calculated for the CIEL*a*b*1976 color space before and after the restoration, and with their color-difference image. In the analysis of this detail—as the gamut of the colors used by the artist was dramatically reduced after the fire of 1897 and by the previous restoration, it is evident that the color parameters have changed with different extents depending on the areas of the paintings. As an example, three spots were highlighted and their colorimetric values were calculated from the data cube (Table 1). They were chosen in order to show the chromatic effect of the restoration procedure on three colors, which are affected in a different manner by the presence of a yellowish aged varnish. Furthermore, this effect, which can be visually appreciated, has to be documented by using a CIE colorimetric approach. To better visualize the results on the painting after the removal of the aged varnish, CIE b* value can be used; it represents the blue–yellow component, with yellow and blue in the positive and negative directions, respectively. In this case, as expected, the bluish area (point 1) reduced the amount of yellow, while the red and flesh tone areas (points 2 and 3, respectively) increased this value resulting after the restoration more yellow (and red) than before. In addition, a* and L* values increased at different levels once the work was finished.

The visualization of these colorimetric variations through a new elaborated image difference is an important tool provided to conservators and art historians to understand the extent the restoration has changed the work.

## 5. Application of a New Compact and Portable HSI System

**Figure 3:**
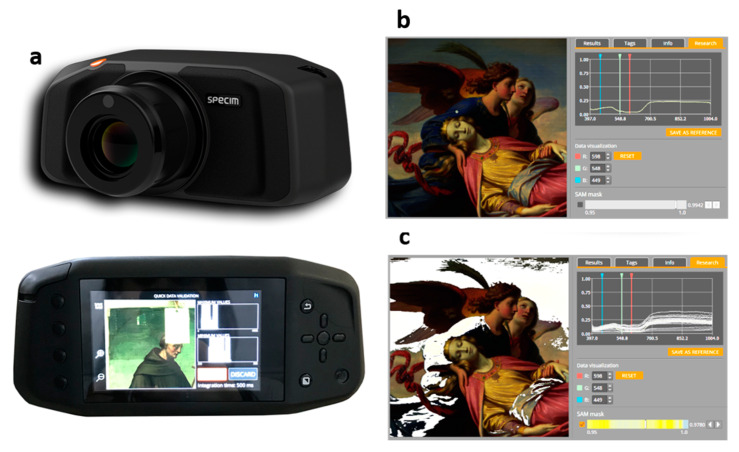
(**a**) Specim IQ hyper-spectral camera; (**b**) image displayed in the camera’s screen with the pixel’s reflectance spectrum; (**c**) cobalt blue distribution map provided by the SAM algorithm in the camera software (in white) on the painting’s surface (figure modified from [60]).

The relatively new Specim IQ HSI camera differs from other HSI devices available on the market since it integrates into a single compact and portable device housing the hyper-spectral sensor, additional color cameras, flash-memory card for data storage. Furthermore, this system works like a reflex camera with an interactive and touch display in the back of the camera that allow controlling the entire set of data acquisition and processing operations (Figure 3). Moreover, it has been designed as a mobile, hand-held and stand-alone HSI camera for applications in different kinds of environments [31,58]. The integrated color camera supports the spectral camera usage by allowing the visualization of the scene and adjustment of the manual focus of the spectral camera.

The camera can be used equally well indoors or outdoors and it works both with natural and artificial light. Like the majority of hyper-spectral cameras, Specim IQ is a push-broom system that makes a line-scan over the target area. The scanning is performed with internal mechanisms. The acquisition process usually takes from a few seconds upwards, so the camera has to be mounted on a tripod.

Specim IQ technical characteristics are the following: 400–1000 nm working range; 7 nm spectral resolution; 3.5 nm spectral sampling; 204 spectral bands; 512 × 512 pixels resulting image; unprocessed and processed data of each are directly saved by the camera and occupied approximately 300 MB. The hyper-spectral data are visualized immediately after the measurement and the user can add metadata to them “Saint Catherine carried by the Angels on Mount Sinai” (49.6 cm × 67 cm, private collection, 19th century, oil painting on canvas) by the Austrian artist Karl von Blaas (1815–1894) is here used as an example of the way in which Specim IQ can be applied to the study of artworks.

The HSI data served to identify the pigments the artist had used and to map them with Spectral Angle Mapping (SAM) algorithm over a particular area. The SAM procedure is a spectral classification tool which is uploaded in the camera’s software. The obtained results revealed, for instance, that the artist had used Thénard’s blue pigment (also known as cobalt blue, an important synthetic pigment used since the 19th century) in the blue and bluish areas. The image that appeared in the camera’s screen can be seen in Figure 3b, in which the graph at the right indicates the pixel extracted from the blue right sleeve of the angel. The identification of the blue pigment as cobalt blue was based on the entire spectral shape and its absorption features in the 500–700 nm range [59]. Subsequently, its areal distribution was mapped with the SAM tolerance thresholding option that was selected from the camera SAM mask tool (Figure 3c). This map was obtained by using a spectrum selected from the blue area of the imaged scene as reference.

## 6. Conclusions

After almost two decades since HSI was slowly introduced in the CH field, this technique has proven its versatility and utility for investigating different typologies of artworks, providing element to answer a variety of conservative issues. The HSI technique demonstrated enormous potentialities, which are partly still unexploited, in particular, in the examination of paintings and works on paper. This imaging technique offers great potential for future developments. In recent years, new application trends are emerging, including applications to photographic materials and an accurate color analysis as a support for restoration interventions. Moreover, new models of user-friendly HSI devices featuring portability and compactness are spreading the use of HSI techniques to several contexts and different environments. All these can contribute to creating other innovative HSI methods and applications and leading to new directions in the study of CH objects.

Finally, HSI is finding its place among other techniques as a preliminary approach to the study of artworks, specifically because it can analyze the entire surface of any nearly flat object.

## Figures and Tables

**Table 1:** Colorimetric values on three selected spots (average of 5 × 5 pixel, spectral acquisition sampled by 2) calculated from the spectra extracted by the cube-files acquired before (L*_b_, a*_b_, b*_b_) and after (L*_a_, a*_a_, b*_a_) the restoration process. CIE standard 2° observer and D65 illuminant, CIEDE2000 color-difference formula [57].

Spot	L*b	L*a	∆L*	a*b	a*a	∆a*	b*b	b*a	∆b*	∆E00
1	27.26	28.05	0.79	2.91	4.17	1.26	6.14	1.99	−4.15	4.24
2	32.62	35.06	2.44	27.82	34.97	7.15	20.66	29.20	8.54	4.64
3	23.65	42.94	19.29	8.89	14.33	5.44	9.56	31.65	22.09	19.58

Coordinates of the spots in the image: (1) x = 250, y = 239; (2) x = 280, y = 310; (3) x = 340, y = 415.
